# Dietary Alaska pollack protein improves skeletal muscle weight recovery after immobilization-induced atrophy in rats

**DOI:** 10.1371/journal.pone.0217917

**Published:** 2019-06-14

**Authors:** Mina Fujitani, Takafumi Mizushige, Fuminori Kawabata, Keisuke Uozumi, Machi Yasui, Kohsuke Hayamizu, Kenji Uchida, Shinji Okada, Bhattarai Keshab, Taro Kishida

**Affiliations:** 1 Laboratory of Nutrition Science, Division of Applied bioscience, Graduate School of Agriculture, Ehime University, Matsuyama, Japan; 2 Department of Applied Biological Chemistry, Faculty of Agriculture, Utsunomiya University, Minemachi, Utsunomiya, Tochigi, Japan; 3 Physiology of Domestic Animals, Faculty of Agriculture and Life Science, Hirosaki University, Bunkyo-cho, Hirosaki, Aomori, Japan; 4 Laboratory of Food Chemistry, Yokohama University of Pharmacy, Yokohama, Japan; 5 Food Function R&D Center, Nippon Suisan Kaisha, Ltd., Tokyo, Japan; 6 Graduate School of Agricultural and Life Sciences, the University of Tokyo, Tokyo, Japan; 7 Food and Health Sciences Research Centre, Graduate School of Agriculture, Ehime University, Matsuyama, Japan; University of California, Davis, UNITED STATES

## Abstract

The promotion of muscle recovery after immobilization is important to preserve an optimum health status. Here, we examined the effect of dietary Alaska pollack protein (APP) on skeletal muscle weight after atrophy induced by hind limb immobilization using plaster immobilization technique. Rat left limb was casted with a wetted plaster cast under anesthesia. After 2 weeks of feeding, the cast was removed and the rats were divided into three groups, namely, a baseline group, high-fat casein diet group, and high-fat APP diet group. After 3 weeks of feeding, the skeletal muscles (soleus, extensor digitorum longus [EDL], and gastrocnemius) were sampled. The estimated weight gains of soleus, gastrocnemius, and EDL muscle in the immobilized limbs were significantly larger in the rats fed with APP diet as compared with those fed with casein diet. In soleus muscle, dietary APP increased the expression of *Igf1* and *Myog* genes in the immobilized limbs after the recovery period.

## Introduction

A number of studies have found that unloading or reduced muscle activity induces significant muscle atrophy [1, 2]. Although skeletal muscle has an inherent capacity to recover from atrophy, the maintenance and recovery of muscle mass and function after disuse sometimes can be slow, inefficient and incomplete [3, 4]. Resistance exercise is not always relevant in specific physio-pathological situations. Thus, the advancement of alternative approaches to promote muscle recovery following disuse is important. Dietary approaches for the positive effects on muscle recovery after immobilization have been examined. Martin *et*.*al*. reported that recovery kinetics varied between diets and the diet supplemented with whey proteins promoted faster recovery of isometric force and concentric power output than a casein-rich diet using plaster immobilization technique, although muscle weight change was not observed [5]. Magne *et*.*al*. and Savary-Auzeloux *et al*. reported that leucine supplementation to diet increased muscle weight in similar models [6, 7].

Fish protein is consumed worldwide. Alaska pollack (*Theragra chalcogramma*) is found in processed seafoods such as imitation crab, kamaboko (fish cakes), and fish sausage. Given the widespread use of fish protein, elucidation of the nutritional characteristics of Alaska pollack is important. In a previous study, we have demonstrated the ability of dietary Alaska pollack protein (APP) to increase gastrocnemius muscle weight in young male Sprague-Dawley rats fed with a high-fat diet as compared with casein after 4 weeks [8]. Furthermore, APP also significantly increased fast-twitch muscle weight, reduced liver triglycerides and serum glucose, and increased gastrocnemius and extensor digitorum longus (EDL) muscle weight after 6 or 8 weeks of feeding [9]. These results suggest that the differences in the quality of proteins may affect muscle weight. In the present study, we examined the effect of dietary APP on muscle atrophy in rats.

The balance between protein synthesis and degradation maintains the skeletal muscle mass. The muscles become hypertrophic as muscle synthesis exceeds decomposition. This balance is mainly controlled by the signals originating from insulin-like growth factor 1 (IGF1) [10]. IGF1 signaling upregulates protein synthesis via protein kinase B (AKT)/mechanistic target of rapamycin (mTOR) pathway and downregulates protein degradation through the suppression of the expression of ubiquitin ligase F-box protein 32 (Fbxo32, also known as atrophy gene-1 [atrogin-1]) and tripartite motif-containing 63 (Trim63, also known as muscle-specific RING finger protein 1 [MuRF1]) [10]. Myostatin is a negative regulator of anabolic pathways that inhibit AKT activation in the skeletal muscle [10]. In addition, the expression of myogenic regulatory factors (MRFs) appears to be regulated in part by the mitotic and myogenic activities of the locally produced IGF1, which functions in an autocrine/paracrine mode [11, 12]. There are substantial evidences to suggest that MRFs are involved in the adaptation of adult muscle fibers in rats [13–17].

In this study, we evaluated the effect of APP on skeletal muscle weight after atrophy induced by hind limb immobilization using plaster immobilization technique. We also evaluated the effect of APP on the expression of *Igf1* and *Mstn* (gene encoding myostatin), ubiquitin ligases, and MRFs after atrophy.

## Materials and methods

### Animals

Male Sprague-Dawley rats (SLC, Shizuoka, Japan) at 5 weeks of age were raised in stainless wire mesh cages in a room controlled by a 12 h light-dark cycle (dark phase: 15:00–3:00) and at a constant temperature (24°C ± 1°C). The animals were separately housed for 6 days for acclimatization to the environment. Animals were fed with regular tap water and a high-fat casein diet, which was formulated using 200 g/kg of casein, 3 g/kg of cystine, 257 g/kg of α-corn starch, 200 g/kg of sucrose, 30 g/kg of soybean oil, 215 g/kg of lard, 50 g/kg of cellulose, 35 g/kg of AIN-93G mineral mixture, and 10 g/kg of AIN-93 vitamin mixture containing 25 g of choline bitartrate/100 g ad libitum during acclimatization. This study was conducted in accordance with the ethical guidelines of the Ehime University Animal Experimentation Committee and was in complete compliance with the National Institutes of Health: Guide for the Care and Use of Laboratory Animals. All efforts were made to minimize the number of animals used and to limit experimentation to what was necessary to produce reliable scientific information.

### Alaska pollack protein

Alaska pollack fillets (Nippon Suisan Kaisha, Ltd., Tokyo, Japan) were used as the fish protein source in this study. The fillets were freeze-dried and ground. The fat component was extracted using hot ethanol (65°C, 60 min × 2) and removed by centrifugation. The remaining ethanol was dried in a vacuum dryer (60°C, about 24 h). Nutritional analyses of casein and fish protein sources were performed at Japan Food Research Laboratories (Tokyo) as previously reported [8, 9]. APP contains almost the same level of protein as casein (Cas, 85.6%; APP, 86.9%) [9].

### Experimental protocols

**Fig 1:**
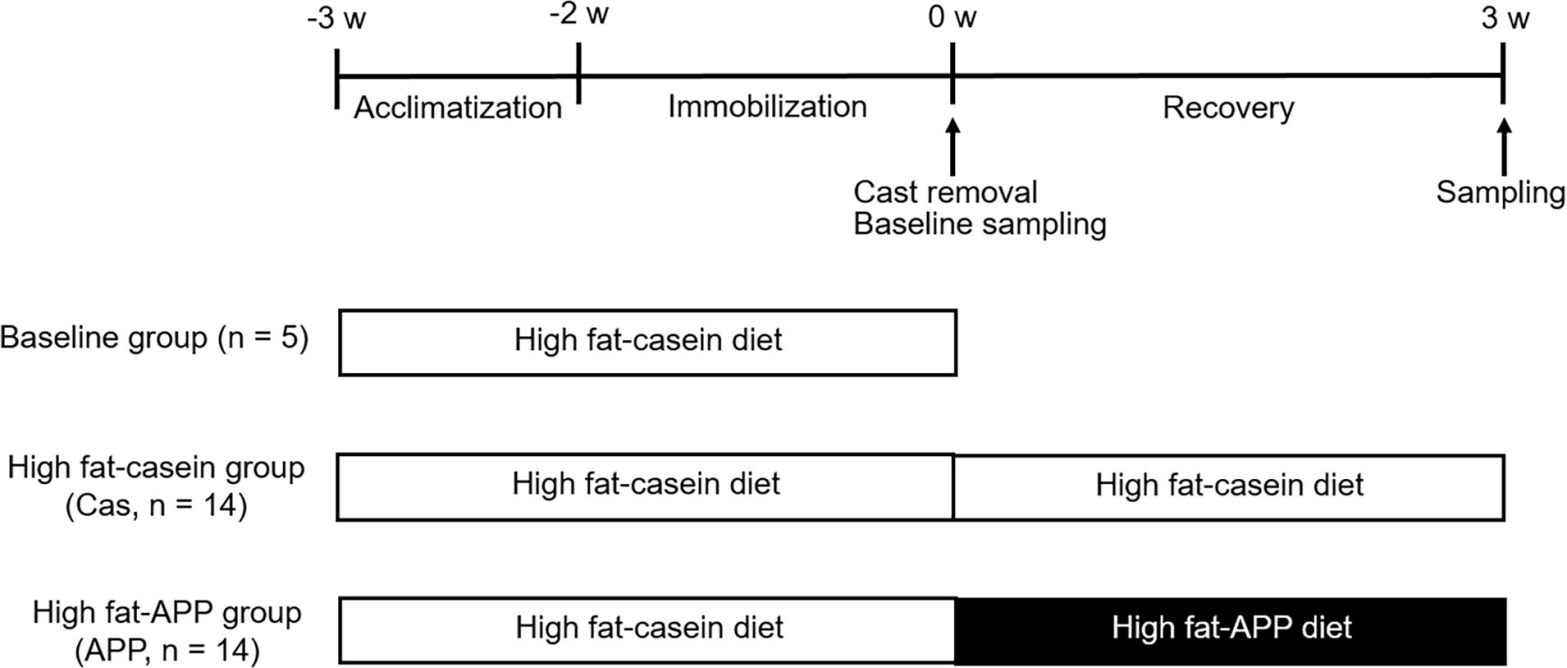
Schedule of rat feeding during immobilization and recovery periods. Left limb was immobilized at knee joint by a plaster and the rats were fed with high-fat casein diet for 2 weeks. After cast removal, five rats were sacrificed (baseline group). The rats were fed with either high-fat casein diet (Cas, n = 14) or high-fat APP diet (APP, n = 14) for the recovery period of 3 weeks and then sacrificed.

Fig 1 shows the schedule of rat feeding during immobilization and recovery periods. After 6 days of acclimatization, 33 rats were subjected to immobilization under an anesthesia with pentobarbital (0.05 mL/100 g body weight); left limbs of rats were splinted by a bended plastic plate (length 50 mm, width 7 mm, bent at a right angle in center with convex facing outward, hand-made from 50 mL of polypropylene centrifuge tube), casted with 45 cm of a wetted plaster cast (3M Soft Cast Casting Tape, width 2.5 cm, 3M Japan, Tokyo, Japan), and coiled and wrapped with a steel wire (diameter 0.45 mm) and steal wire mesh (diameter 0.45 mm, 1 × 1 mm opening) to inhibit gnaw. After 2 weeks of feeding, the casts were removed and the rats were divided into three groups as follows: baseline group (n = 5), high-fat casein diet group (n = 14), and high-fat APP diet group (n = 14). The rats in high-fat casein group were fed with high-fat casein diet, the same diet during acclimatization, while the rats in APP diet group were fed with a diet that replaced casein with fish protein. During recovery period, the body weight and amount of food consumed were recorded every morning for each animal, and then the food was replenished. After 3 weeks of feeding, the rats in these two experimental groups were killed by decapitation, and blood samples corresponding to a non-fasting state were collected from the neck at 20:00 on the last day of the experimental period. The skeletal muscles of soleus, gastrocnemius, and EDL were removed and weighed. The soleus muscle was stored at −80°C until gene expression analysis. The rats from the baseline group were killed by decapitation just after cast removal and the samples were collected as described above. We failed to obtain the intact EDL muscle from rats from the baseline group.

### Quantitative real-time polymerase chain reaction (RT-qPCR)

As previously described [18], whole soleus muscles were homogenized to isolate total RNA using Sepasol-RNA I Super G (Nacalai Tesque Inc., Kyoto, Japan) following manufacturer’s instruction as previously described, and DNase treated using RNase-Free DNase (Takara Bio, Shiga, Japan) for 30 min at 37°C. RNA purity (260/280 nm ratio) was determined by a spectrophotometer (BioSpectrometer Basic; Eppendorf, Tokyo, Japan), and RNA integrity was checked electrophoretically by 1% agarose-formaldehyde gel stained with ethidium bromide (Sigma-Aldrich Japan K.K., Tokyo, Japan). Messenger RNA (mRNA) was isolated from total RNA using Oligotex-dT30 (Takara Bio, Shiga, Japan). Complementary DNA (cDNA) was synthesized from mRNA using reverse transcriptase (Reverse Transcriptase XL [AMV] for RT-PCR, 5 U/μL, Takara Bio) and Oligo (dt) primer on a thermal cycler (ABI GeneAmp2400, PerkinElmer, Waltham, MA, USA). After cDNA synthesis, RT-qPCR for target genes and endogenous reference gene *Ppia* and *Actb* were run separately, and amplifications were performed with a StepOnePlus real-time PCR system (Applied Biosystems, Foster, CA, USA) by using THUNDERBIRD SYBR qPCR mix (TOYOBO, Osaka, Japan). Melting point dissociation curves were used to confirm the purity of the amplification products. We confirmed that gene expression of *Ppia* was in parallel with that of *Actb*, and selected *Ppia* as housekeeping gene based on the coefficient of variation (SD/mean·100) of the quantification cycle (Cq) values. The basic amplification program was set to perform 50 cycles of denaturation for 15 s at 95°C and annealing and extension for 1 min at 60°C. Fluorescence was recorded at 530 nm during extension. Relative mRNA expression was calculated using the Cq of each target gene with that of the *Ppia* gene as the reference and the corresponding real-time PCR efficiency of respective primer sets by the method previously reported [19]. All primers were designed using online Primer3 software Primer3input (primer3 http://bioinfo.ut.ee/primer3-0.4.0/ v 0.2). Primer sequences are provided in Table 1.

**Table 1:** Primer sequences used in the present study.

Genes		Sequences	Product size (bp)	NCBI ID
*Igf1*	sense	CTTGAGCAACCTGCAAAACA	80	NM_001082477
	antisense	GGAAATGCCCATCTCTGAAA		
*Mstn* (myostatin)	sense	GATGGGCTGAATCCCTTTTT	94	NM_019151
	antisense	CCGTGGAGTGTTCATCACAG		
*Fbxo32* (atrogin-1)	sense	AAGCTTGTGCGATGTTACCC	81	NM_133521
	antisense	CCAGGAGAGAATGTGGCAGT		
*Trim63* (MuRF1)	sense	AGTCGCAGTTTCGAAGCAAT	80	NM_080903
	antisense	CTGCTTCTCCAGGTTCTCCA		
*Myf5*	sense	CCACCTCCAACTGCTCTGAT	90	NM_001106783
	antisense	CAGGGCAGTAGATGCTGTCA		
*Myod1* (MyoD)	sense	GGGGTTCAGGAGTGACAGAA	95	NM_176079
	antisense	CGGCGATAGTAGCTCCATGT		
*Myog* (myogenin)	sense	CCTGCCCTGAGATGAGAGAG	91	NM_017115
	antisense	TGGAAGGTTCCCAATATCCA		
*Myf6* (MRF4)	sense	TGTACCCAGGGAGTGATGGT	89	NM_013172
	antisense	AACGTGTTCCTCTCCACTGC		
*Ppia* (cyclophilin A)	sense	ATGGTCAACCCCACCGTGTT	206	NM_017101
	antisense	CGTGTGAAGTCACCACCCT		

*Igf1*, insulin-like growth factor 1; *Fbxo32*, F-box protein 32; atrogin-1, atrophy gene-1; *Trim63*, tripartite motif-containing 63; MuRF1, muscle-specific RING finger protein-1; *Myf5*, myogenic factor 5; *Myod1*, myogenic differentiation 1; *Myf6*, myogenic factor 6; MRF4, myogenic regulatory factor 4; *Ppia*, peptidylprolyl isomerase A.

### Statistical analysis

Data are expressed as mean ± standard error of mean (SEM). The changes in skeletal muscle mass during the recovery period was calculated according to the following equation. Δskeletal muscle mass = (skeletal muscle mass at week3)—(skeletal muscle mass at week0). Statistical analysis was performed with Student’s paired *t*-test for skeletal muscles weights in baseline group. For other measurement factors, statistical analysis was performed with Student’s unpaired *t*-test. Statistical significance was defined as *P* < 0.05. All statistical analyses were performed using the IBM SPSS Statistics software package (SPSS Japan Inc., IBM, Tokyo, Japan).

## Results

**Fig 2:**
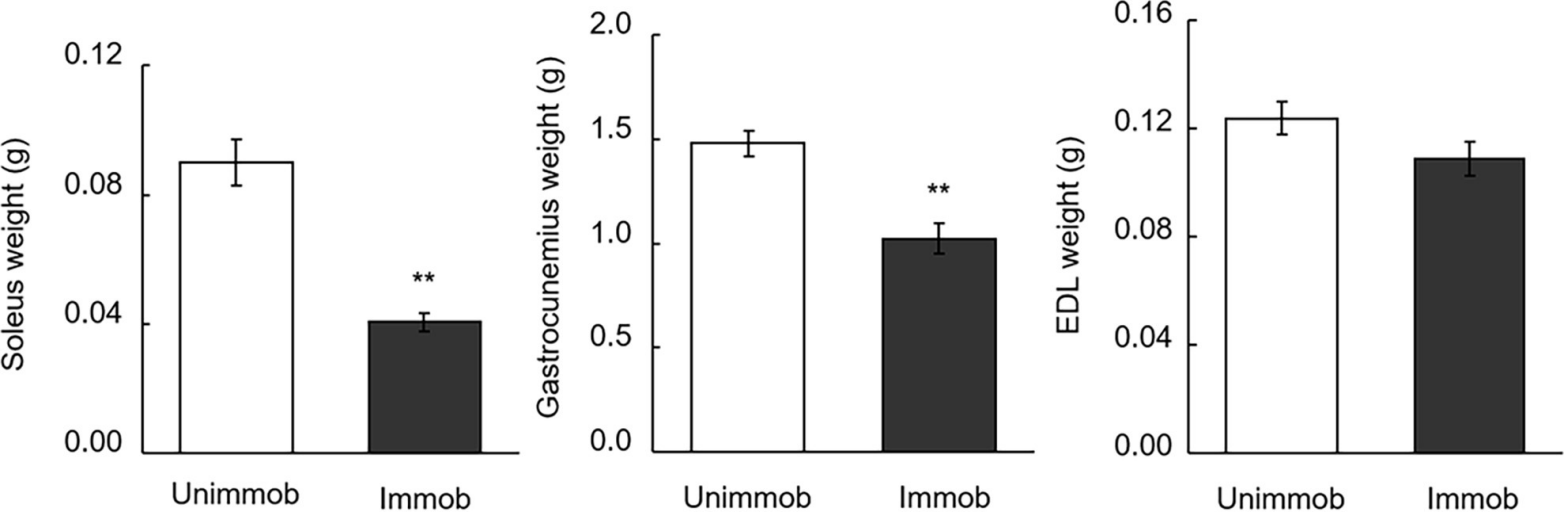
Skeletal muscle weights in baseline group. The weights of soleus, gastrocnemius, and extensor digitorum longus (EDL) muscles in unimmobilized (Unimmob) and immobilized (Immob) limbs from baseline group (n = 4–5) after the immobilization period are shown. Data are expressed as mean ± standard error of mean (SEM). Asterisks indicate significant differences as compared to unimmobilized limbs by Student’s paired *t*-test. **P < 0.01.

**Fig 3:**
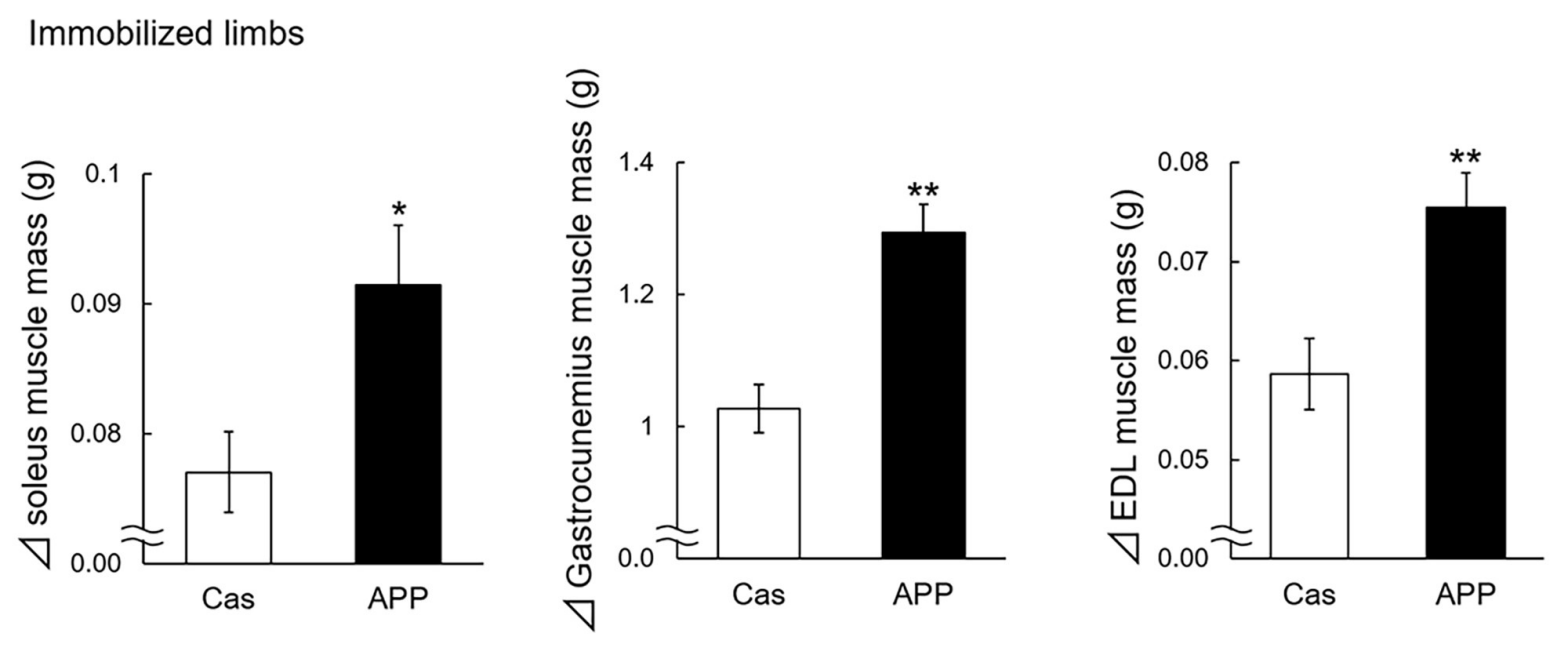
Estimated skeletal muscle weight gains in immobilized limbs during recovery period. The estimated weight gains of soleus, gastrocnemius, and extensor digitorum longus (EDL) muscles in immobilized limbs of high-fat casein diet group (Cas, n = 14) and high-fat APP diet group (APP, n = 14) during the recovery period are shown. The gains in skeletal muscle weight during the recovery period were estimated according to the following equation: Δskeletal muscle weight = (skeletal muscle weight at week 3 in Cas or APP group)—(average skeletal muscle weight at week 0 in baseline group). Data are expressed as mean ± standard error of mean (SEM). Statistical analysis was performed between Cas and APP group with the Student’s unpaired *t*-test. *P < 0.05, **P < 0.01.

The weights of skeletal muscles were compared between unimmobilized and immobilized limbs in the baseline group at week 0 (Fig 2). Gastrocnemius and soleus muscle weights significantly decreased by immobilization. Estimated skeletal muscle weight gain during the recovery period of 3 weeks were compared between high-fat casein diet group and high-fat APP diet group to evaluate the dietary APP effect on muscle recovery. The estimated weight gains of soleus, gastrocnemius, and EDL muscle in the immobilized limbs were significantly larger in the rats fed with APP diet as compared with those fed with casein diet (19%, 26%, and 29% larger, respectively) (Fig 3). These three muscle weights in the immobilized limbs after recovery were significantly larger in the rats fed with APP diet as compared with those fed with casein diet (Table 2). Food intake, energy intake, and protein intake, of the rats fed with APP diet were significantly lower than that of the rats fed with casein diet, although no significant difference was observed between APP and casein groups in body weight gain (Table 2).

**Table 2:** Effects of dietary APP on food intake, protein intake, energy intake, body weight and skeletal muscle weight during the recovery period.

		Cas			APP		
	
Food intake	g/3 w	408	±	6	377	±	6**
Protein intake	g/3 w	70	±	1	66	±	1**
Energy intake	MJ/3 w	8.3	±	0.1	7.7	±	0.1**
Initial body weight	g	258	±	3	257	±	3
Final body weight	g	402	±	5	406	±	5
Body weight gain	g/3 w	144	±	4	149	±	3
Soleus muscle weight	g	0.118	±	0.003	0.13	±	0.003**
Gastrocnemius muscle weight	g	2.050	±	0.04	2.316	±	0.04**
EDL muscle weight	g	0.167	±	0.004	0.184	±	0.004**

Food intake, protein intake, energy intake, initial body weight, final body weight, body weight gain, and soleus, gastrocnemius and EDL muscle weight in immobilized limbs of high-fat casein diet group (Cas, n = 14) and high-fat APP diet group (APP, n = 14) during the recovery period are shown. Data are expressed as means ± standard errors (SEM). Statistical analysis was performed with the Student’s unpaired *t*-test.

**P < 0.01.

Quantitative PCR analysis revealed the changes in gene expression in soleus muscle after 3 weeks of recovery following dietary APP supplementation. Dietary APP significantly increased *Igf1* and *Myog* (gene encoding myogenin) expression in immobilized limbs as compared with casein group (Table 3).

**Table 3:** Effects of dietary APP on gene expression of the regulators involved in catabolism, anabolism, and myogenic process in immobilized limbs after recovery period.

		Cas			APP		
		target gene mRNA/*ppia* mRNA					
*Igf1*	(×10^−1^)	2.90	±	0.26	3.94	±	0.36*
*Mstn* (myostatin)	(×10^−3^)	0.83	±	0.07	0.91	±	0.11
*Fbxo32* (atrogin-1)	(×10^−2^)	0.36	±	0.03	0.41	±	0.05
*Trim63* (MuRF1)	(×10^−2^)	0.40	±	0.05	0.38	±	0.04
*Myf5*	(×10^−2^)	1.10	±	0.06	1.23	±	0.09
*Myod1* (MyoD)	(×10^−3^)	4.75	±	1.50	6.44	±	1.02
*Myog* (Myogenin)	(×10^−1^)	0.50	±	0.02	0.62	±	0.04*
*Myf6* (MRF4)	(×10^−1^)	0.61	±	0.03	0.62	±	0.05

Igf1, Mstn (myostatin), Fbxo32 (atrogin-1), Trim63 (MuRF1), Myf5, Myod1 (MyoD), Myog (myogenin) and Myf6 (MRF4) gene expression of soleus muscle in immobilized limbs of high-fat casein diet group (Cas, n = 14) and high-fat APP diet group (APP, n = 14) after recovery period is shown. Data are expressed as means ± standard error of mean (SEM). Statistical analysis was performed with the Student’s unpaired t-test.

*P < 0.05. Igf1, insulin-like growth factor 1; Fbxo32, F-box protein 32; atrogin-1, atrophy gene-1; Trim63, tripartite motif-containing 63; MuRF1, muscle-specific RING finger protein-1; Myf5, myogenic factor 5; Myod1, myogenic differentiation 1; Myf6, myogenic factor 6; Myog, myogenin; MRF4, myogenic regulatory factor 4.

## Discussion

In our previous studies, dietary APP tended to increase gastrocnemius muscle weight without immobilization after 4 weeks of feeding with no significant difference and significantly increased gastrocnemius and EDL muscle weights after 8 weeks of feeding [8, 9]. In the present study, after immobilization, dietary APP intake significantly increased the estimated weight gains of in gastrocnemius, soleus, and EDL muscles as compared with casein intake during 3 weeks of the recovery period (Fig 3). This suggests that the effect of dietary APP on skeletal muscle would be strong in the recovery of atrophy than in the normal state. Although dietary APP significantly increased the estimated weight gains of these three muscle weights in contralateral unimmobilized limbs (S1 Fig), it is difficult to evaluate the stimulatory effects of APP on skeletal muscle in unimmobilized limbs because unimmobilized limbs could be over worked or otherwise effected by the immobilization of the other limb. The result suggests that dietary APP may exert the stimulatory effects on skeletal muscle weights, regardless of the degree of muscle disuse atrophy before its supplementation. Dietary APP supplementation may be an alternative approach to stimulate muscle recovery for patients being unable to perform physical exercise.

The present study revealed that dietary APP significantly increased the gene expression of *Igf1* in immobilized limbs after 3 weeks of reambulation (Table 3), consistent with the increase in muscles weight. A similar trend was observed in contralateral unimmobilized limbs (*P* = 0.116, unpaired *t*-test) (S1 Table). However, it is difficult to evaluate the effects of APP on *Igf1* expression in unimmobilized limbs because unimmobilized limbs could be over worked or otherwise effected by the immobilization of the other limb. IGF1 acts as an anabolic factor and functions in an autocrine/paracrine mode in the skeletal muscle [11]. Signaling pathways downstream of IGF1 are important for the control of the balance between protein synthesis and degradation [10]. IGF1 signaling upregulates protein synthesis via protein kinase AKT / mTOR pathway and downregulates protein degradation through the suppression of the expression of ubiquitin ligase *Fbxo32* and *Trim63* [10]. Myostatin is a potent autocrine/paracrine negative regulator of anabolic pathways that inhibits AKT activation in skeletal muscle [10, 20]. In the present study, dietary APP had no effect on *Fbxo32*, *Trim63*, and *Mstn* gene expression in immobilized and unimmobilized limbs (Table 3). It is speculated that IGF binding proteins may have interfered with the effects of IGF-I on *Fbxo32*, *Trim63*, and *Mstn* gene expression. One of these potential intercalators is IGFBP-5, which is the major IGFBP secreted by skeletal muscle. Stevens-Lapsley *et*.*al*. reported that IGFBP-5 mRNA was significantly higher after 1 week of reambulation in tibialis anterior muscles [21]. However, the increase in muscles weight were consistent with significantly increased the gene expression of *Igf1* in the rats fed with APP diet after 3 weeks of reambulation in the present study (Fig 3 and Table 3). We suggest that the enhanced IGF1 signaling after dietary APP supplementation may increase muscle weights not through the suppression of protein degradation but through the promotion of protein synthesis. However, we did not confirm the effect of dietary APP on protein synthesis in skeletal muscle in the present study. Further studies are warranted to determine the level of suppression of protein degradation and promotion of protein synthesis necessary to contribute for muscle hypertrophy induced by dietary APP.

Dietary APP significantly increased the expression of *Myog* but not other MRFs in immobilized after 3 weeks of reambulation (Table 3), consistent with the increase in muscle weight. A similar trend was observed in contralateral unimmobilized limbs (*P* = 0.079, unpaired *t*-test) (S1 Table). However, it is difficult to evaluate the effects of APP on *Myog* expression in unimmobilized limbs because unimmobilized limbs could be over worked or otherwise effected by the immobilization of the other limb. A hierarchical relationship between the four MRFs was defined by the temporal specificity of the expression of each family member. The progression of the activated satellite cells toward the myogenic lineage is mainly controlled by the expression of Myf5 and Myod1. As myoblasts initiate the process of differentiation toward myotubes, high level expression of Myod1 results in the expression of myogenin and downregulation of Myf5 expression. This switch in the expression pattern from Myf5 to myogenin coincides with cell cycle exit and a commitment to the differentiation process. In mature muscle fibers, the expression of Myod1 and myogenin is downregulated [22, 23]. Myf6 continues to be expressed at high levels and acts as a predominant MRF in adult muscles [23], although it serves as a negative regulator of muscle growth [24]. The autocrine/paracrine functions of IGF1 may stimulate muscle regeneration and hypertrophy through the induction of both proliferation and differentiation of myoblasts and satellite cells [12]. A correspondence between elevated IGF1 expression and increased *Myog* gene expression and other myogenic gene products was observed using C_2_C_12_ cells [25]. Stimulation of autocrine/paracrine IGF1 signaling and the subsequent increase in *Myog* expression may be one of the reasons underlying the stimulatory effects of dietary APP on skeletal muscle weights.

Very little information is available on the active ingredients in APP. Branched-chain amino acids, especially leucine, may potentially promote skeletal muscle [26]. However, APP used in the present study contains almost the same level of leucine and other branched-chain amino acids as casein [9]. The arginine content of APP is twice as much as casein [9]. Dietary arginine supplementation shifts nutrient partitioning to promote muscle gain over fat gain [27]. In addition, the amount of cystine and glycine in APP is thrice the amount present in casein, while its aspartic acid content is one and a half times higher. There is no information available about the relationship between these amino acids and skeletal muscle promotion, presumably through stimulation of autocrine/paracrine IGF1 signaling and the subsequent increase in *Myog* expression.

Surprisingly, despite significant increases in skeletal muscle weights and no significant differences in body weight gain, significant decreases in energy intake and protein intake observed in rats fed with APP diet in the present study. Although the reasons for this are unclear, a number of previous studies have reported that amino acid composition, rate of absorption, and protein/food texture may be important factors for protein-stimulated metabolic effects [28]. It is possible that amino acid composition, rate of absorption, and protein/food texture of APP are responsible for decreases in ad libitum energy intake and protein intake.

Important questions remain to be answered about the hypertrophic effect of dietary APP on skeletal muscle weights. First, we examined the effect of dietary APP on skeletal muscle weight in rats fed with high-fat diet. In our previous studies, we used high-fat diet to evaluate anti-obesity or anti-hyperlipidemia effects and found promoting effects of APP on skeletal muscle weight. Therefore, we designed the present study using high-fat diet feeding. Whether high-fat diet feeding is essential to observe the effect of APP is an important question that needs to be addressed in future studies. Second, in the present study, we did not examine the effects of dietary APP supplementation on skeletal muscle weights during the period of immobilization. Further studies are needed to determine whether dietary APP supplementation is a new approach to ameliorate muscle disuse atrophy.

In conclusion, we found that soleus, gastrocnemius, and EDL muscle weight significantly increased in the rats fed with APP diet as compared with the rats fed with casein diet in the recovery period after immobilization both in immobilized and unimmobilized limbs. Taken together with our previous findings [8, 9], dietary APP may exert the stimulatory effects on skeletal muscle weights, regardless of the degree of muscle disuse atrophy before its supplementation. It is possible that dietary APP supplementation may be a new approach to stimulate muscle recovery after immobilization-induced atrophy. In soleus muscle, dietary APP increased *Igf1* and *Myog* gene expression in soleus muscle in immobilized and unimmobilized limbs. Stimulation of autocrine/paracrine IGF1 signaling and the subsequent increase in *Myog* expression may be one of reasons underlying the positive effects of dietary APP on skeletal muscle weight.

## Supporting information

S1 Fig Estimated skeletal muscle weight gains in contralateral unimmobilized limbs during recovery period. The estimated weight gains of soleus, gastrocnemius, and extensor digitorum longus (EDL) muscles in contralateral unimmobilized limbs (Unimmob) limbs of high-fat casein diet group (Cas, n = 14) and high-fat APP diet group (APP, n = 14) during the recovery period are shown. The gains in skeletal muscle weight during the recovery period were estimated according to the following equation: Δskeletal muscle weight = (skeletal muscle weight at week 3 in Cas or APP group)—(average skeletal muscle weight at week 0 in baseline group). Data are expressed as mean ± standard error of mean (SEM). Statistical analysis was performed with the Student’s unpaired t-test. *P < 0.05, **P < 0.01.
(PDF)

S1 Table Effects of dietary APP on gene expression of the regulators involved in catabolism, anabolism, and myogenic process in contralateral unimmobilized limbs after recovery period. *Igf1*, *Mstn* (myostatin), *Fbxo32* (atrogin-1), *Trim63* (MuRF1), *Myf5*, *Myod1* (MyoD), *Myog* (myogenin) and *Myf6* (MRF4) gene expression of soleus muscle in contralateral unimmobilized limbs of high-fat casein diet group (Cas, n = 14) and high-fat APP diet group (APP, n = 14) after recovery period is shown. Data are expressed as means ± standard error of mean (SEM). Statistical analysis was performed with the Student’s unpaired *t*-test. There was no significant difference between Cas and APP. *Igf1*, insulin-like growth factor 1; *Fbxo32*, F-box protein 32; atrogin-1, atrophy gene-1; *Trim63*, tripartite motif-containing 63; MuRF1, muscle-specific RING finger protein-1; *Myf5*, myogenic factor 5; *Myod1*, myogenic differentiation 1; *Myf6*, myogenic factor 6; *Myog*, myogenin; MRF4, myogenic regulatory factor 4.
(PDF)
