# Supplementary material for: Dietary Alaska pollack protein improves skeletal muscle weight recovery after immobilization-induced atrophy in rats
Source: PLoS One. 2019 Jun 14;14(6):e0217917. doi: 10.1371/journal.pone.0217917 (PMC6570023; doi:10.1371/journal.pone.0217917)
Supplement: S1 Fig — The estimated weight gains of soleus, gastrocnemius, and extensor digitorum longus (EDL) muscles in contralateral unimmobilized limbs (Unimmob) limbs of high-fat casein diet group (Cas, n = 14) and high-fat APP diet group (APP, n = 14) during the recovery period are shown. The gains in skeletal muscle weight during the recovery period were estimated according to the following equation: Δskeletal muscle weight = (skeletal muscle weight at week 3 in Cas or APP group)—(average skeletal muscle weight at week 0 in baseline group). Data are expressed as mean ± standard error of mean (SEM). Statistical analysis was performed with the Student’s unpaired t-test. *P < 0.05, **P < 0.01. (PDF) [file pone.0217917.s001.pdf]

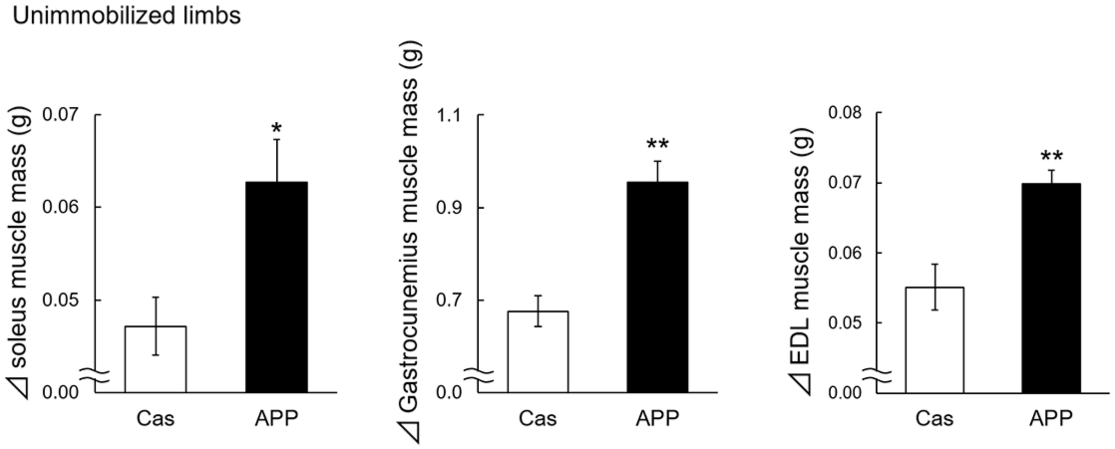

S1 Fig. Estimated skeletal muscle weight gains in contralateral unimmobilized limbs during recovery period. The estimated weight gains of soleus, gastrocnemius, and extensor digitorum longus (EDL) muscles in contralateral unimmobilized limbs (Unimmob) limbs of high-fat casein diet group (Cas, n = 14) and high-fat APP diet group (APP, n = 14) during the recovery period are shown. The gains in skeletal muscle weight during the recovery period were estimated according to the following equation: Δskeletal muscle weight = (skeletal muscle weight at week 3 in Cas or APP group) - (average skeletal muscle weight at week 0 in baseline group). Data are expressed as mean ± standard error of mean (SEM). Statistical analysis was performed with the Student's unpaired t-test. *P < 0.05, **P < 0.01.
