# Supplementary material for: Dietary Alaska pollack protein improves skeletal muscle weight recovery after immobilization-induced atrophy in rats
Source: PLoS One. 2019 Jun 14;14(6):e0217917. doi: 10.1371/journal.pone.0217917 (PMC6570023; doi:10.1371/journal.pone.0217917)
Supplement: S1 Table — Igf1, Mstn (myostatin), Fbxo32 (atrogin-1), Trim63 (MuRF1), Myf5, Myod1 (MyoD), Myog (myogenin) and Myf6 (MRF4) gene expression of soleus muscle in contralateral unimmobilized limbs of high-fat casein diet group (Cas, n = 14) and high-fat APP diet group (APP, n = 14) after recovery period is shown. Data are expressed as means ± standard error of mean (SEM). Statistical analysis was performed with the Student’s unpaired t-test. There was no significant difference between Cas and APP. Igf1, insulin-like growth factor 1; Fbxo32, F-box protein 32; atrogin-1, atrophy gene-1; Trim63, tripartite motif-containing 63; MuRF1, muscle-specific RING finger protein-1; Myf5, myogenic factor 5; Myod1, myogenic differentiation 1; Myf6, myogenic factor 6; Myog, myogenin; MRF4, myogenic regulatory factor 4. (PDF) [file pone.0217917.s002.pdf]

**S1 Table. Effects of dietary APP on gene expression of the regulators involved in catabolism, anabolism, and myogenic process in contralateral unimmobilized limbs after recovery period.**

| | | Cas | APP |
|---|---|---|---|
| | | target gene mRNA/*ppia* mRNA | |
| *Igf1* | ($\times 10^{-1}$) | 3.54 ± 0.30 | 4.15 ± 0.23 |
| *Mstn* (myostatin) | ($\times 10^{-3}$) | 2.11 ± 0.27 | 2.02 ± 0.21 |
| *Fbxo32* (atrogin-1) | ($\times 10^{-2}$) | 1.13 ± 0.12 | 0.93 ± 0.13 |
| *Trim63* (MuRF1) | ($\times 10^{-2}$) | 1.35 ± 0.09 | 1.25 ± 0.14 |
| *Myf5* | ($\times 10^{-2}$) | 0.75 ± 0.05 | 0.73 ± 0.08 |
| *Myod1* (MyoD) | ($\times 10^{-3}$) | 1.25 ± 0.28 | 1.48 ± 0.30 |
| *Myog* (Myogenin) | ($\times 10^{-1}$) | 1.26 ± 0.05 | 1.48 ± 0.11 |
| *Myf6* (MRF4) | ($\times 10^{-1}$) | 1.52 ± 0.11 | 1.51 ± 0.15 |

*Igf1*, *Mstn* (myostatin), *Fbxo32* (atrogin-1), *Trim63* (MuRF1), *Myf5*, *Myod1* (MyoD), *Myog* (myogenin) and *Myf6* (MRF4) gene expression of soleus muscle in contralateral unimmobilized limbs of high-fat casein diet group (Cas, n = 14) and high-fat APP diet group (APP, n = 14) after recovery period is shown. Data are expressed as means ± standard error of mean (SEM). Statistical analysis was performed with the Student's unpaired *t*-test. There was no significant difference between Cas and APP. *Igf1*, insulin-like growth factor 1; *Fbxo32*, F-box protein 32; atrogin-1, atrophy gene-1; *Trim63*, tripartite motif-containing 63; MuRF1, muscle-specific RING finger protein-1; *Myf5*, myogenic factor 5; *Myod1*, myogenic differentiation 1; *Myf6*, myogenic factor 6; *Myog*,

myogenin; MRF4, myogenic regulatory factor 4.
